# Large language models are comparable with commonly used statistical software: A validation of GPT 5.1 for frequentist meta‐analysis in orthopaedics

**DOI:** 10.1002/ksa.70379

**Published:** 2026-03-11

**Authors:** Mikhail Salzmann, Nikolai Ramadanov, Robert Prill, Robert Hable, Roland Becker

**Affiliations:** ^1^ Centre of Orthopaedics, Traumatology and Plastic Surgery, Brandenburg Medical School University Hospital Brandenburg an der Havel Brandenburg an der Havel Germany; ^2^ Faculty of Health Science Brandenburg Brandenburg Medical School Theodor Fontane Brandenburg an der Havel Germany; ^3^ Faculty of Applied Computer Science Deggendorf Institute of Technology Deggendorf Germany

**Keywords:** artificial intelligence, heterogeneity, meta‐research, orthopaedic research, statistical validation

## Abstract

**Purpose:**

The purpose of this study was to evaluate whether Chat Generative Pre‐trained Transformer (ChatGPT; Version 5.1) can reproduce frequentist meta‐analytic calculations with an accuracy comparable to established statistical software in orthopaedic research.

**Methods:**

In this methodological comparison study, data from two previously published orthopaedic meta‐analyses with identical statistical architectures as reference standards were used. Between‐study variance (*τ* ^2^) was estimated using the Sidik–Jonkman method and uncertainty was quantified using the Hartung–Knapp adjustment for the random‐effects models, while common‐effect models assume *τ* ^2^ = 0. Original data extraction tables were provided to ChatGPT‐5.1, which was instructed to perform the same analyses. ChatGPT‐generated pooled mean differences, confidence intervals and heterogeneity statistics (*I* ^2^, *τ* ^2^, *p* values) were compared with verified reference results obtained using the meta and metafor packages in R.

**Results:**

Across seven evaluated outcomes, ChatGPT‐5.1 reproduced the direction of effects in all cases. Deviations compared with reference meta‐analyses were classified as minor in three outcomes (43%), moderate in one outcome (14%) and major in three outcomes (43%). Agreement was highest in low‐heterogeneity settings, whereas substantial deviations occurred in outcomes with pronounced between‐study heterogeneity, particularly under random‐effects models.

**Conclusion:**

ChatGPT‐5.1 demonstrates emerging capability to approximate frequentist meta‐analytic calculations, particularly in low‐heterogeneity settings. However, its tendency to underestimate between‐study variability and to deviate in complex random‐effects scenarios limits its reliability as a standalone tool. At present, large language models may support exploratory analyses but cannot fully replace dedicated statistical software for meta‐analyses in orthopaedic research.

**Level of Evidence:**

Level III.

**Abbreviations**
AI: artificial intelligence
AMSTAR: Assessing the Methodological Quality of Systematic Reviews
CEF: common effects model
ChatGPT: Chat Generative Pre‐trained Transformer
CI: confidence interval
FAI: femoroacetabular impingement syndrome
HHS: Harris Hip Score
HOS‐ADL: Hip Outcome Score–Activities of Daily Living
iHOT: International Hip Outcome Tool
LLM: large language model
MCID: minimum clinically important difference
RCT: randomized controlled trial
REF: random effects model
RoB: risk of bias
SD: standard deviation

## INTRODUCTION

Systematic reviews and meta‐analyses of primary studies are a fundamental aspect of evidence‐based medicine [11, 20]. Studies in orthopaedics often show a high heterogeneity in methodology, patient populations and outcome measures [14, 19, 24]. However, well‐performed meta‐analyses have the power to substantially contribute to clinical guidelines, structured clinical decision making and to inform new research [12, 23]. Despite their value, conducting a meta‐analysis can be highly demanding, as data extraction requires a thorough understanding of clinical and methodological context, just as statistical analysis demands specialized statistical expertise. As a result, researchers rely on specialized statistical software or collaborate with professional statisticians to ensure statistical correctness [5].

Recent literature has reported about integrating artificial intelligence (AI), particularly large language models (LLMs) such as the Chat Generative Pre‐trained Transformer (ChatGPT), in scientific workflows such as literature search [7], academic writing [1, 4], abstract screening and even conducting systematic reviews [13, 25]. However, these findings are not without limitations, including mixed results regarding overall efficacy and reliability—with some studies reporting substantial underperformance compared with human raters [8]—as well as documented instances of hallucination phenomena during literature searches [3]. In this context, hallucinations describe the generation of plausible‐sounding but factually incorrect or unverifiable information by LLMs, particularly when tasks exceed their capacity for formal algorithmic or statistical validation. These limitations currently prevent a fully automated use of AI and make its application feasible only with substantial and continuous human oversight.

The ability of ChatGPT to perform statistical calculations for meta‐analyses remains unclear in the literature, especially in Orthopaedics. If ChatGPT is capable of conducting such analyses accurately, it may have the potential to fundamentally change how meta‐analyses are performed in the future. Therefore, the aim of the present study was to compare the ability of ChatGPT in its current version (Version 5.1) with commonly used statistical software. It was hypothesized that ChatGPT 5.1 is able to perform statistical calculations as accurate as specialized statistical software.

## METHODS

### Ethical considerations

This meta‐research study is based exclusively on data extracted from previously published meta‐analyses with their related primary studies. All original studies received institutional ethical approval and informed consent according to local regulations. As no individual patient identifiers were used and no new data were collected, additional ethical approval for this secondary analysis was not required.

### Study design

This is a methodological comparison study. The data pool of two previously published meta‐analyses [15, 17] from our author group was used as reference material. One meta‐analysis investigated postoperative Harris Hip Score (HHS) outcomes following hip replacement surgery across multiple follow‐up intervals, while the second evaluated functional outcomes following conservative treatment versus hip arthroscopy for femoroacetabular impingement syndrome (FAIS). Both analyses were based exclusively on randomized controlled trials (RCTs) and reported continuous outcomes using identical frequentist statistical frameworks. In the original publications, all statistical analyses had been conducted by a professional statistician (R. H.) using the packages meta (Guido Schwarzer) and metafor (Wolfgang Viechtbauer) in R (R Foundation for Statistical Computing), which served as the methodological gold standard for comparison.

#### Selection of reference meta‐analyses

Our author group has published numerous meta‐analyses across different methodological frameworks, including frequentist standard models, indirect comparison analyses, network meta‐analyses and multilevel approaches, using a variety of heterogeneity estimators and weighting strategies. For the present methodological evaluation, two previously published meta‐analyses [15, 17] were selected because they shared an identical statistical architecture and therefore allowed a controlled and internally consistent comparison. Both meta‐analyses applied a frequentist framework with inverse‐variance weighting and reported results under both common‐effect and random‐effects assumptions. Between‐study variance (*τ* ^2^) was estimated using the Sidik–Jonkman method and uncertainty was quantified using the Hartung–Knapp adjustment [16] for the random‐effects models, while common‐effect models assume *τ* ^2^ = 0. This methodological homogeneity ensured that differences between published results and ChatGPT‐5.1 outputs could be attributed to the model's computational behaviour rather than to heterogeneity in analytical design. Because heterogeneity metrics were extracted directly from the reference meta‐analyses, constellations with low or zero *I* ^2^ and non‐zero *τ* ^2^ may occur and reflect estimator‐specific behaviour rather than computational inconsistency.

#### Statistical framework and model definitions

In this study, a frequentist meta‐analytic framework was applied using inverse‐variance weighting. Two effect models were considered throughout: a common‐effect model and a random‐effects model. The common‐effect model assumes a single true underlying effect across studies and therefore sets the between‐study variance to *τ* ^2^ = 0. In contrast, the random‐effects model allows for between‐study heterogeneity, with *τ* ^2^ estimated using the Sidik–Jonkman method [16] and uncertainty quantified via the Hartung–Knapp adjustment. Both models were reported in the reference meta‐analyses and were reproduced accordingly for comparison with the ChatGPT‐5.1 outputs.

#### Quality appraisal of the reference meta‐analyses

Both selected meta‐analyses [15, 17] were appraised using the Assessing the Methodological Quality of Systematic Reviews (AMSTAR)‐2 tool [22] to ensure that their methodological procedures were adequately documented and compatible with use as reference standards. The appraisal followed the 16‐item AMSTAR‐2 framework, focusing on protocol availability, literature search methods, study selection and data extraction processes, risk‐of‐bias assessment and the statistical approach to quantitative synthesis. No AMSTAR‐2 results are reported here, as the appraisal served solely to verify methodological suitability for inclusion in this comparison study. Quality appraisal was performed independently by two orthopaedic researchers (N.R. and M.S.). Any discrepancies were resolved through discussion until consensus was reached.

#### Data preparation

To enable a direct comparison between the published results and the ChatGPT‐5.1 output, key summary metrics from the forest plots of the two reference meta‐analyses were extracted, including number of studies, mean differences with confidence intervals (CIs) and heterogeneity measures (*I* ^2^, *τ* ^2^ and *p*‐value). Data extraction was performed independently by two orthopaedic researchers (N.R. and M.S.). Any discrepancies were resolved through discussion until consensus was reached. For the ChatGPT‐5.1 analysis, the original data extraction tables prepared for the statistician were reformatted into a standardized structure including group allocation, sample size, outcome values and corresponding standard deviations. ChatGPT‐5.1 was instructed to perform a frequentist meta‐analysis using predefined methodological specifications, including common‐effect and random‐effects models under inverse‐variance weighting, with Sidik–Jonkman *τ* ^2^ estimation and Hartung–Knapp adjustment applied to the random‐effects models. The full structure of the input tables is provided in Tables [Link], [Link], [Link], [Link], [Link], [Link], [Link], as well as the complete wording instructions (Table S9) to ensure reproducibility.

#### Human oversight and validation

All ChatGPT‐5.1 outputs were compared against the effect estimates reported in the two reference meta‐analyses [15, 17]. To ensure that these published values were correctly extracted and interpreted, all reference results (effect sizes, CIs and heterogeneity metrics) were verified against the published values and independently recalculated in R (R Foundation for Statistical Computing), again using the packages meta (Guido Schwarzer) and metafor (Wolfgang Viechtbauer), solely for validation purposes. No new statistical analyses were conducted. The full statistical code and model specifications underlying the reference meta‐analyses are available in the original publications [15, 17]. Deviations between ChatGPT‐generated results and the verified reference values were classified into three categories: (1) minor deviations (rounding‐ or precision‐related); (2) moderate deviations (misinterpretation of input or formula structure) and (3) major errors (incorrect model specification, weighting or heterogeneity estimation). All disagreements were reviewed jointly by two orthopaedic researchers (N. R. and M. S.) and one statistician (R.H.) and final classifications were reached by consensus.

## RESULTS

### Description of the two reference meta‐analyses

The two reference meta‐analyses [15, 17] used in this comparison applied a comparable frequentist framework with inverse‐variance weighting and identical analytical architecture. Both implemented the Hartung–Knapp–Sidik–Jonkman approach for variance estimation and reported pooled mean differences under common‐effect and random‐effects assumptions. The 2024 meta‐analysis [17] evaluated postoperative HHS versus HHS difference in RCTs of hip replacement, synthesizing results from 41 RCTs (3572 patients) to compare minimally invasive versus conventional surgical approaches. The 2025 meta‐analysis [15] examined outcomes of conservative treatment versus hip arthroscopy for FAIS, pooling seven RCTs (973 patients) and reporting functional minimum clinically important difference (MCID), international Hip Outcome Tool (iHOT) and Hip Outcome Score–Activities of Daily Living (HOS‐ADL) as continuous endpoints. Both meta‐analyses provided complete summary metrics, including pooled mean differences with CIs and heterogeneity measures (*I* ^2^, *τ* ^2^, *p* values), allowing their use as standardized reference datasets for comparison with ChatGPT‐5.1 outputs.

### Quality appraisal of the two reference meta‐analyses

Both reference meta‐analyses [15, 17] were appraised using the AMSTAR‐2 framework (Table 1). Both reviews demonstrated clear PICO definitions, preregistered PROSPERO protocols, comprehensive multi‐database search strategies, duplicate screening and extraction procedures and structured risk‐of‐bias assessments using the Cochrane RoB‐2 tool. Both analyses applied consistent frequentist statistical methods, including inverse‐variance weighting, Hartung–Knapp adjustment and the Sidik–Jonkman estimator, reporting results under common‐effect and random‐effects assumptions. Heterogeneity metrics (*I* ^2^, *τ* ^2^ and *Q* tests) and publication bias assessments were provided. While both studies fulfilled all critical AMSTAR‐2 domains for quantitative synthesis, neither review systematically incorporated the risk‐of‐bias assessments into the interpretation of results, which is classified as a non‐critical AMSTAR‐2 domain. No funding bias or conflicts of interest affecting methodological quality were identified. A complete AMSTAR‐2 appraisal of the two reference meta‐analyses is provided in the Supporting Information (Table S8).

**Table 1:** AMSTAR‐2 appraisal of the two reference meta‐analyses used as comparator datasets.

AMSTAR‐2 critical domain	Meta‐analysis 2024 [17]	Meta‐analysis 2025 [15]
Protocol registered before commencement (Item 2)	Yes	Yes
Adequate literature search (Item 4)	Yes	Yes
Study selection performed in duplicate (Item 5)	Yes	Yes
Data extraction performed in duplicate (Item 6)	Yes	Yes
Adequate risk‐of‐bias assessment (Item 9)	Yes	Yes
Appropriate statistical methods used (Item 11)	Yes	Yes
Consideration of RoB in interpretation (Item 13)	Partially reported	Partially reported
Assessment of publication bias (Item 15)	Yes	Yes

Abbreviations: AMSTAR‐2, Assessing the Methodological Quality of Systematic Reviews‐2; RoB, risk of bias.

### Reproduction of meta‐analytic results by ChatGPT‐5.1 and agreement with the two reference meta‐analyses

Across all outcomes extracted from the two reference meta‐analyses [15, 17], ChatGPT‐5.1 was able to reproduce the direction and magnitude of effects with varying numerical agreement (Table 2). For the FAIS meta‐analysis [15], ChatGPT closely matched the published pooled mean differences for the post‐intervention functional MCID and the iHOT ≤ 12 months outcomes under both common‐effect and random‐effects assumptions, with only minor deviations in the CI boundaries. Heterogeneity statistics (*I* ^2^, *τ* ^2^, *p* value) also showed near‐identical patterns, except for small differences in *τ* ^2^ due to rounding and internal precision. For HOS‐ADL ≤ 8 months, ChatGPT reproduced the pooled effects of both models almost exactly, including the high heterogeneity observed in the original analysis.

**Table 2:** Reproduction of pooled effect estimates and heterogeneity statistics by ChatGPT‐5.1 in comparison with the two reference meta‐analyses.

Study	Outcome	Model type	Between‐study heterogeneity estimator	Weighting method	Number of sturdies	Mean difference	CIs	Heteroegeneity *I* ^2^	Heteroegeneity *τ* ^2^	Heteroegeneity *p*
Ramadanov et al. [15]	Post‐intervention functional MCID	CEF	Sidik‐Jonkman heterogeneity estimator with HK adjustment	Inverse variance	7	0.85	0.53–1.17	0.00	0.02	0.96
		REF				0.85	0.64–1.06			
Chat GPT		CEF				0.85	0.72–0.98	0.00	0.00	0.97
		REF				0.85	0.66–1.04			
Ramadanov et al. [15]	iHOT <12 months	CEF			6	10.74	7.06–14.42	0.00	7.52	0.62
		REF				10.98	6.62–15.34			
Chat GPT		CEF				10.75	7.10–14.41	0.00	0.00	0.61
		REF				10.75	6.68–14.83			
Ramadanov et al. [15]	HOS‐ADL < 8 months	CEF			3	5.62	1.76–9.48	69.00	29.88	0.04
		REF				4.10	−12.31 to 20.50			
Chat GPT		CEF				5.62	1.76–9.48	0.00	30.19	0.37
		REF				4.09	−12.32 to 20.50			

Study	Outcome	Model type	Between‐study heterogeneity estimator	Weighting method	Number of sturdies	Mean difference	CI	Heteroegeneity *I* ^2^	Heteroegeneity *τ* ^2^	Heteroegeneity *p*
Ramadanov et al. [17]	HHS 0–0.5 months postop	CEF	Sidik–Jonkman heterogeneity estimator with HK adjustment	Inverse variance	25	5.12	4.85–5.38	95.00	21.52	<0.01
		REF				5.14	3.17–7.11			
Chat GPT		CEF				4.51	4.25–4.67	29.60	11.61	0.04
		REF				3.33	1.93–4.73			
Ramadanov et al. [17]	HHS 3 months postop	CEF			25	2.50	2.22–2.78	93.00	16.77	<0.01
		REF				3.34	1.51–5.17			
Chat GPT		CEF				2.26	1.99–2.53	16.20	8.09	0.18
		REF				2.06	0.91–3.21			
Ramadanov et al. [17]	HHS 6 months postop	CEF			19	1.19	0.85–1.54	86.00	10.40	<0.01
		REF				2.56	0.79–4.33			
Chat GPT		CEF				0.93	0.62–1.24	22.50	2.67	0.10
		REF				1.15	0.34–1.97			
Ramadanov et al. [17]	HHS 12 months postop	CEF			17	0.90	0.44–1.35	8.00	1.70	0.36
		REF				1.11	0.35–1.87			
Chat GPT		CEF				0.51	0.17–0.86	0.00	0.00	0.99
		REF				0.51	0.24–0.78			

*Note*: *I* ^2^ and *τ* ^2^ are reported as published in the reference meta‐analyses; differences between both metrics reflect their relative (*I* ^2^) versus absolute (*τ* ^2^) nature and estimator‐specific properties.

Abbreviations: CEF, common effect model; CI, confidence interval; HHS, Harris Hip Score; HK, Hartung–Knapp; HOS‐ADL, Hip Outcome Score–Activities of Daily Living; iHOT, International Hip Outcome Tool; MCID, Minimal Clinically Important Difference; REF, random effects model.

For the hip replacement meta‐analysis [17], ChatGPT reproduced the direction of effects for all postoperative HHS endpoints (≤1.5, 3, 6 and 12 months), but tended to generate more conservative pooled estimates and narrower CIs under the common‐effect model. Under the random‐effects model, ChatGPT produced estimates that remained within the same clinical range but deviated more substantially from the published pooled values, particularly for early postoperative HHS (0–1.5 months). Heterogeneity metrics were also reproduced directionally, though ChatGPT consistently returned lower *I* ^2^ and *τ* ^2^ values, indicating a tendency to underestimate between‐study variability.

Across the seven evaluated outcomes, deviations between ChatGPT‐5.1 and the reference meta‐analyses were classified as minor in three outcomes (43%), moderate in one outcome (14%) and major in three outcomes (43%). Minor deviations were primarily observed in low‐heterogeneity settings (*I* ^2^ < 30%), whereas major deviations occurred predominantly in outcomes with substantial between‐study heterogeneity and higher Sidik–Jonkman *τ* ^2^ estimates. Overall, ChatGPT showed high agreement for outcomes with low heterogeneity and smaller sample sizes, and moderate agreement for outcomes with substantial heterogeneity and wide between‐study variance, as reflected in the published Sidik–Jonkman estimates (Table 2).

## DISCUSSION

The most important finding of this methodological study is that ChatGPT‐5.1 is generally able to perform statistical calculations for frequentist meta‐analyses as accurate as specialized statistical software. However, these results should be interpreted with caution, as substantial deviations in the calculation of individual parameters were observed in selected cases. A highly controlled analytical framework was established by selecting two meta‐analyses with identical methodological designs to investigate the model's behaviour. This model relies generally on strict instructions on how calculations are performed, such as the common‐effects and random‐effect model selection or the heterogeneity estimator. Whereas in most cases, calculations of the GPT model were almost identical to the gold standard, in some cases, the model showed a higher deviation. The highest deviation has been observed in the calculation of statistical heterogeneity, where the model partly presented incomprehensive results with the tendency of underestimating between‐study variability. A not further investigated tendency was observed, that the model experienced increasing difficulties as the number of included studies and analytical parameters increased. The observed deviations are likely attributable to the inherent architecture of LLMs. Unlike statistical software, GPT‐5.1 does not execute predefined algorithms but generates outputs based on probabilistic pattern recognition. While this approach appears robust for low‐variance and low‐heterogeneity data, it may lead to simplified internal approximations when complex variance structures, large numbers of studies or multi‐step estimators such as Sidik–Jonkman with Hartung–Knapp adjustment are required. Future developments integrating explicit computational backends or hybrid AI–statistical frameworks may improve numerical stability and reproducibility.

### Performance of ChatGPT 5.1

Across low‐heterogeneity outcomes—such as MCID and iHOT measures in the FAIS meta‐analysis, GPT‐5.1 performed remarkably well. The LLM yielded pooled mean differences that were nearly identical to those obtained using R, with only minor deviations in CI boundaries. The high level of concordance was observed under both common‐effect and random‐effects assumptions. These findings are consistent with emerging evidence that LLMs can handle structured, low‐variance numerical tasks with increasing accuracy, provided the input data are clearly defined and methodological instructions are explicit [13, 25]. In contrast, outcomes characterized by substantial between‐study heterogeneity—most notably early postoperative HHS values in the hip replacement meta‐analysis—revealed notable discrepancies. GPT‐5.1 tended to underestimate *τ* ^2^ and *I* ^2^ and produced narrower CIs than the reference models. This pattern suggests that GPT‐5.1 may apply internal approximations or simplified heuristics when confronted with highly variable data, despite having explicit instructions to implement Sidik–Jonkman estimation with Hartung–Knapp adjustment. Similar observations have been described in recent research showing that LLMs are prone to reduced accuracy when tasks require multi‐step numerical reasoning or the application of specialized statistical estimators [8].

### Sources of deviation and methodological implications

The observed deviations stem primarily from GPT‐5.1 inherent architecture. LLMs do not execute code or run statistical algorithms in the traditional sense. They generate outputs based on patterns learned during training. Even though GPT‐5.1 has been optimized for analytical reasoning, its internal decision pathways remain opaque and may not fully replicate formal statistical procedures, particularly when confronted with complex variance structures or large sample sizes [18]. Ultimately, this ‘black box’ phenomenon is a well‐known problem of pre‐trained neuronal networks in general, with previously reported difficulties in the interpretation of results generated by AI [21].

### Comparison with existing literature

This limitation aligns with previous findings on LLM applications in evidence synthesis. Studies evaluating ChatGPT's ability to conduct systematic reviews or meta‐analyses have reported promising results but also highlighted risks of miscalculation, misinterpretation of study characteristics and algorithmic drift when tasks exceed simple pattern recognition [8, 13, 24]. Importantly, GPT‐based approaches have been shown to exhibit hallucinations in literature searches [3] and inconsistencies in methodological steps requiring precise algorithmic instructions [7]. Our study further contributes to this growing body of evidence, demonstrating that even when provided with correctly extracted data and precise methodological instructions, GPT‐5.1 may diverge from reference calculations. While the model performed well in low‐heterogeneity scenarios, it showed reduced reliability when variance estimation played a central analytical role. This has direct implications for clinical specialties, such as orthopaedics, where heterogeneity is frequently substantial due to variations across surgical techniques, rehabilitation protocols and outcome measurement tools [14, 19, 24]. Other researchers have reported consistent results, for example, in performing statistical analysis in epidemiological or biomedical studies, highlighting also a much higher user‐friendliness of ChatGPT compared to professional statistical software, thus potentially lowering the boundaries for researchers to conduct complex statistical analysis [6, 9].

Despite its limitations, GPT‐5.1 demonstrated a promising capacity to approximate formal meta‐analytic calculations, particularly for low‐heterogeneity datasets. This capability may be useful for rapid prototyping or initial exploratory analyses. However, given the persistent risk of numerical drift, underestimation of heterogeneity and deviations in variance estimation, AI‐generated statistical outputs cannot yet replace conventional statistical software in clinical research. Continuous human oversight remains essential, as highlighted by previous literature on AI‐supported systematic reviews [3, 8, 13, 25].

Future developments integrating explicit computational engines or embedded statistical modules may improve the reproducibility and mathematical reliability of LLM‐based analyses and could ultimately enable the execution of even complex meta‐analyses within a very short time. This would optimize the currently time‐consuming process to such an extent that the synthesized evidence is less likely to become outdated by the time the meta‐analysis is completed [2, 10].

## STRENGTHS AND LIMITATIONS

A major strength of our study is the controlled analytical environment created by selecting two methodologically identical reference meta‐analyses. This allowed us to isolate deviations arising from the LLM itself rather than differences in meta‐analytic framework. Additionally, data were re‐verified using R to ensure that all reported values were accurate.

However, there are also several limitations to our study. (i) The model was evaluated under highly controlled conditions with predefined and explicit statistical instructions. Therefore, the observed performance reflects an idealized experimental setting and may differ substantially from unsupervised real‐world use. (ii) Varying instructions might have influenced results. Alternative or more detailed instructions might yield different accuracy or even different results. (iii) Two meta‐analyses have been used for our study. Upscaling the number of reported outcomes might have relativized or even changed the evaluation of mathematical accuracy. (iv) It should be noted that the published literature has primarily evaluated earlier and now outdated versions of ChatGPT, such as GPT‐4. Therefore, direct comparability with the present findings is limited. (v) Another important aspect not addressed in the present study is intra‐model variability. The reproducibility of GPT‐5.1 outputs when identical complex meta‐analytic tasks are repeated across multiple runs remains unclear and warrants systematic investigation in future studies.

## CONCLUSION

GPT‐5.1 shows emerging potential for supporting meta‐analytic workflows in orthopaedic research, but it does not currently demonstrate the mathematical reliability required to replace dedicated statistical software. Its performance is strongest in well‐controlled low‐heterogeneity settings and weakest in complex random‐effects scenarios. AI can currently augment but not automate statistical synthesis, at least under present technological conditions. Thus, ChatGPT‐5.1 should be regarded as an exploratory support tool rather than an analytical replacement: while it may assist in hypothesis generation or preliminary analyses, final meta‐analytic results must still rely on dedicated statistical software and expert oversight.

## AUTHOR CONTRIBUTIONS

All authors contributed to the study conception and design. Material preparation and data collection were performed by Mikhail Salzmann and Nikolai Ramadanov. Analysis and statistics were performed by Mikhail Salzmann, Nikolai Ramadanov, Robert Hable and Robert Prill. The first draft of the manuscript was written by Mikhail Salzmann, Nikolai Ramadanov and Roland Becker. All authors commented on previous versions of the manuscript. All authors read and approved the final manuscript.

## CONFLICT OF INTEREST STATEMENT

The authors declare no conflicts of interest.

## ETHICS STATEMENT

The authors have nothing to report.

## Supporting information

Supplementary Table 1. Exemplary data extraction table provided to ChatGPT‐5.1 for meta‐analytic calculation.

Supplementary Table 2. Exemplary data extraction table provided to ChatGPT‐5.1 for meta‐analytic calculations.

Supplementary Table 3. Exemplary data extraction table provided to ChatGPT‐5.1 for meta‐analytic calculations.

Supplementary Table 4. Exemplary data extraction table provided to ChatGPT‐5.1 for meta‐analytic calculations.

Supplementary Table 5. Exemplary data extraction table provided to ChatGPT‐5.1 for meta‐analytic calculations.

Supplementary Table 6. Exemplary data extraction table provided to ChatGPT‐5.1 for meta‐analytic calculations.

Supplementary Table 7. Exemplary data extraction table provided to ChatGPT‐5.1 for meta‐analytic calculations.

Supplementary Table 8. Complete AMSTAR‐2 appraisal of the two reference meta‐analyses.

Supplementary 9. Complete wording instructions to the language model.

## Data Availability

The datasets generated and/or analysed during the current study are available from the corresponding authors upon request. Relevant data supporting the findings of this study are also provided in the Supporting Information.
