# Supplementary material for: Large language models are comparable with commonly used statistical software: A validation of GPT 5.1 for frequentist meta‐analysis in orthopaedics
Source: Knee Surg Sports Traumatol Arthrosc. 2026 Mar 11;34(8):3002–10. doi: 10.1002/ksa.70379 (PMC13418392; doi:10.1002/ksa.70379)
Supplement: Supplementary file 8 — Supplementary Table 8. Complete AMSTAR‐2 appraisal of the two reference meta‐analyses. [file KSA-34-3002-s001.docx]

| AMSTAR-2 Item | Description | Meta-analysis 2024 [17] | Meta-analysis 2025 [15] |
| --- | --- | --- | --- |
| 1 | PICO clearly defined | Yes | Yes |
| 2 | Protocol registered before review | Yes | Yes |
| 3 | Justification for study designs | Yes | Yes |
| 4 | Comprehensive literature search | Yes | Yes |
| 5 | Study selection in duplicate | Yes | Yes |
| 6 | Data extraction in duplicate | Yes | Yes |
| 7 | List of excluded studies with reasons | Yes | Yes |
| 8 | Description of included studies | Yes | Yes |
| 9 | Risk-of-bias assessment | Yes (RoB 2) | Yes (RoB 2) |
| 10 | Adequate meta-analytic methods | Yes (HK-SJ, CE/RE models, IV weighting) | Yes (HK-SJ, CE/RE models, IV weighting) |
| 11 | Heterogeneity assessment | Yes (I², τ², Q-test) | Yes (I², τ², Q-test) |
| 12 | RoB considered in interpretation | Partially | Partially |
| 13 | Explanation for study funding | Yes | Yes |
| 14 | Appropriate synthesis of individual studies | Yes | Yes |
| 15 | Publication bias assessed | Yes | Yes |
| 16 | Conflict of interest reported | Yes | Yes |
