# Supplementary material for: Large language models are comparable with commonly used statistical software: A validation of GPT 5.1 for frequentist meta‐analysis in orthopaedics
Source: Knee Surg Sports Traumatol Arthrosc. 2026 Mar 11;34(8):3002–10. doi: 10.1002/ksa.70379 (PMC13418392; doi:10.1002/ksa.70379)
Supplement: Supplementary file 9 — Supplementary 9. Complete wording instructions to the language model. [file KSA-34-3002-s006.docx]

The specific instruction provided to ChatGPT-5.1 was as follows: “Using the data table provided (Column A = intervention group coded as 1 or 2, Column B = sample size, Column C = continuous outcome measure [HHS/iHOT/HOS-ADL], Column D = standard deviation), perform a frequentist meta-analysis. Apply both a common-effects and a random-effects model using inverse-variance weighting with the Sidik–Jonkman heterogeneity estimator and Hartung–Knapp adjustment. Report the pooled mean difference with 95% confidence intervals, and provide heterogeneity statistics (I², τ², p-value). In addition, methodological specifications from the original meta-analyses were extracted to guide the model: model type (common vs random effects), heterogeneity estimator (Sidik–Jonkman), and weighting method (inverse variance). ChatGPT-5.1 was then prompted to perform a frequentist meta-analysis using the supplied table, applying the prespecified model type, estimator, and weighting method, and to report mean differences with confidence intervals and heterogeneity statistics (I², τ², p-value).
